# *N*,*P*,*P*-Triisopropyl­phosphinic amide

**DOI:** 10.1107/S1600536811018551

**Published:** 2011-05-20

**Authors:** Normen Peulecke, Bhaskar R. Aluri, Bernd H. Müller, Anke Spannenberg, Uwe Rosenthal

**Affiliations:** a Leibniz-Institut für Katalyse e. V. an der Universität Rostock, Albert-Einstein-Strasse 29A, 18059 Rostock, Germany

## Abstract

The title compound, C_9_H_22_NOP, was obtained by slow diffusion of oxygen into a toluene solution of ^i^Pr_2_PNH^i^Pr. In the crystal, an inter­molecular N—H⋯O hydrogen bond occurs.

## Related literature

For the synthesis of the starting compound (^i^Pr)_2_PNH^*i*^Pr, see: Kuchen *et al.* (1990 ▶). For a similar synthesis of the title compound, see: Brück *et al.* (1995 ▶). For similar structures of *R*_2_P(O)NH*R* in which the P atom has at least one attached alkyl substituent, see: Burns *et al.* (1997 ▶); Denmark & Dorow (2002 ▶); Kolodiazhnyi *et al.* (2003 ▶); Francesco *et al.* (2010 ▶).
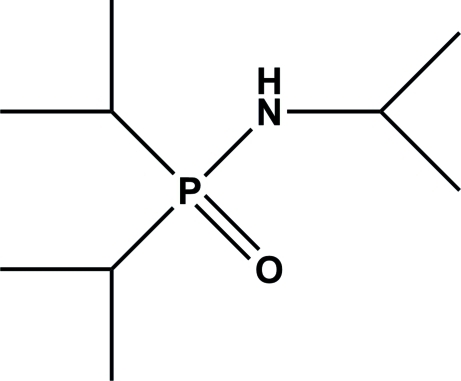


## Experimental

### 

#### Crystal data

C_9_H_22_NOP
*M* *_r_* = 191.25
Monoclinic, 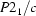

*a* = 15.030 (3) Å
*b* = 8.4813 (17) Å
*c* = 10.071 (2) Å
β = 107.36 (3)°
*V* = 1225.3 (4) Å^3^
*Z* = 4
Mo *K*α radiation
μ = 0.19 mm^−1^
*T* = 195 K
0.42 × 0.26 × 0.20 mm

#### Data collection

Stoe IPDS II diffractometer
19581 measured reflections
2807 independent reflections
2012 reflections with *I* > 2σ(*I*)
*R*_int_ = 0.035

#### Refinement

*R*[*F* ^2^ > 2σ(*F* ^2^)] = 0.039
*wR*(*F* ^2^) = 0.091
*S* = 0.89
2807 reflections
115 parameters
H-atom parameters constrained
Δρ_max_ = 0.39 e Å^−3^
Δρ_min_ = −0.16 e Å^−3^

### 

Data collection: *X-AREA* (Stoe & Cie, 2005 ▶); cell refinement: *X-AREA*; data reduction: *X-AREA*; program(s) used to solve structure: *SHELXS97* (Sheldrick, 2008 ▶); program(s) used to refine structure: *SHELXL97* (Sheldrick, 2008 ▶); molecular graphics: *XP* in *SHELXTL* (Sheldrick, 2008 ▶); software used to prepare material for publication: *SHELXL97*.

## Supplementary Material

Crystal structure: contains datablocks I, global. DOI: 10.1107/S1600536811018551/yk2008sup1.cif

Structure factors: contains datablocks I. DOI: 10.1107/S1600536811018551/yk2008Isup2.hkl

Additional supplementary materials:  crystallographic information; 3D view; checkCIF report

## Figures and Tables

**Table 1:** Hydrogen-bond geometry (Å, °)

*D*—H⋯*A*	*D*—H	H⋯*A*	*D*⋯*A*	*D*—H⋯*A*
N1—H1*A*⋯O1^i^	0.88	1.98	2.8344 (17)	165

Symmetry code: (i) 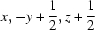.

**Fig. 1.**
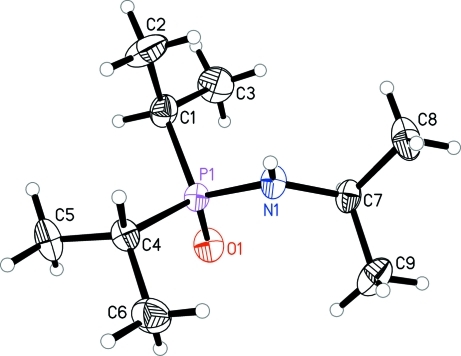
The molecular structure of the title compound showing the atom-labelling scheme. Displacement ellipsoids are drawn at the 30% probability level.

## supplementary crystallographic information

### Comment

Aminophosphines with alkyl-substituents undergo oxidation very easily compared to their analogue aryl-substituted species. Most of the structurally characterized *P*,*P*-diorganylphosphinic amides *R*^1^*R*^2^P(O)NH*R*^3^ have a sterogenic phosphorus or nitrogen centre (Burns *et al.*, 1997, Denmark *et al.*, 2002, Kolodiazhnyi *et al.*, 2003 and Francesco *et al.*, 2010). Here we report about the structural characterization of the known compound (^i^Pr)_2_P(O)N(H)^i^Pr (Fig. 1). The P1—O1 distance is with 1.4799 (11) Å in the range of a P═O double bond. A strong intermolecular hydrogen bond N1—H1A···O1 (N1···O1 2.834 (2), H1A···O1 1.98 Å and N1—H1A···O1 165°) was observed.

### Experimental

A toluene solution (20 mL) of 0.4 g (2.3 mmol) (^i^Pr)_2_PN(H)^i^Pr (Kuchen *et al.*, 1990) was exposed to dry air over a period of 48 h. After evaporation of the solvent, the oily residue was dissolved in n-hexane, filtrated and stored at -40°C for crystallization. After 3 days colourless crystals were formed, which were suitable for X-ray analysis. The analytical data of C_9_H_22_NOP correlated with those in the literature (Brück *et al.*, 1995).

### Refinement

H atoms were placed in idealized positions with d(N—H) = 0.88, d(C—H) = 0.98 (CH_3_) and 1.00 Å (CH) and refined using a riding model with *U*_iso_(H) fixed at 1.5 *U*_eq_(C) for CH_3_ and 1.2 *U*_eq_(C) for NH and CH.

### Crystal data

**Table:**

C_9_H_22_NOP	*F*(000) = 424
*M**_r_* = 191.25	*D*_x_ = 1.037 Mg m^−^^3^
Monoclinic, *P*2_1_/*c*	Mo *K*α radiation, λ = 0.71073 Å
Hall symbol: -P 2ybc	Cell parameters from 5563 reflections
*a* = 15.030 (3) Å	θ = 2.1–29.2°
*b* = 8.4813 (17) Å	µ = 0.19 mm^−^^1^
*c* = 10.071 (2) Å	*T* = 195 K
β = 107.36 (3)°	Prism, colourless
*V* = 1225.3 (4) Å^3^	0.42 × 0.26 × 0.20 mm
*Z* = 4	

### Data collection

**Table:**

Stoe IPDS II diffractometer	2012 reflections with *I* > 2σ(*I*)
Radiation source: fine-focus sealed tube	*R*_int_ = 0.035
graphite	θ_max_ = 27.5°, θ_min_ = 2.8°
ω scans	*h* = −19→19
19581 measured reflections	*k* = −11→11
2807 independent reflections	*l* = −13→13

### Refinement

**Table:**

Refinement on *F*^2^	Primary atom site location: structure-invariant direct methods
Least-squares matrix: full	Secondary atom site location: difference Fourier map
*R*[*F*^2^ > 2σ(*F*^2^)] = 0.039	Hydrogen site location: inferred from neighbouring sites
*wR*(*F*^2^) = 0.091	H-atom parameters constrained
*S* = 0.89	*w* = 1/[σ^2^(*F*_o_^2^) + (0.0547*P*)^2^] where *P* = (*F*_o_^2^ + 2*F*_c_^2^)/3
2807 reflections	(Δ/σ)_max_ = 0.001
115 parameters	Δρ_max_ = 0.39 e Å^−^^3^
0 restraints	Δρ_min_ = −0.16 e Å^−^^3^

### Special details

**Table:**

Geometry. All e.s.d.'s (except the e.s.d. in the dihedral angle between two l.s. planes) are estimated using the full covariance matrix. The cell e.s.d.'s are taken into account individually in the estimation of e.s.d.'s in distances, angles and torsion angles; correlations between e.s.d.'s in cell parameters are only used when they are defined by crystal symmetry. An approximate (isotropic) treatment of cell e.s.d.'s is used for estimating e.s.d.'s involving l.s. planes.
Refinement. Refinement of *F*^2^ against ALL reflections. The weighted *R*-factor *wR* and goodness of fit *S* are based on *F*^2^, conventional *R*-factors *R* are based on *F*, with *F* set to zero for negative *F*^2^. The threshold expression of *F*^2^ > σ(*F*^2^) is used only for calculating *R*-factors(gt) *etc*. and is not relevant to the choice of reflections for refinement. *R*-factors based on *F*^2^ are statistically about twice as large as those based on *F*, and *R*- factors based on ALL data will be even larger.

### Fractional atomic coordinates and isotropic or equivalent isotropic displacement parameters (Å^2^)

**Table:**

	*x*	*y*	*z*	*U*_iso_*/*U*_eq_	
C1	0.70679 (12)	0.5276 (2)	0.6731 (2)	0.0525 (4)	
H1B	0.6513	0.5582	0.5941	0.063*	
C2	0.68976 (19)	0.5848 (2)	0.8073 (3)	0.0830 (7)	
H2A	0.7434	0.5574	0.8868	0.125*	
H2B	0.6336	0.5342	0.8178	0.125*	
H2C	0.6813	0.6995	0.8034	0.125*	
C3	0.79173 (16)	0.6067 (3)	0.6497 (3)	0.0802 (7)	
H3A	0.7812	0.7207	0.6396	0.120*	
H3B	0.8021	0.5645	0.5650	0.120*	
H3C	0.8466	0.5858	0.7294	0.120*	
C4	0.61524 (11)	0.22776 (19)	0.69367 (17)	0.0418 (4)	
H4	0.6119	0.2607	0.7874	0.050*	
C5	0.52745 (12)	0.2872 (3)	0.5848 (2)	0.0653 (6)	
H5A	0.5320	0.2652	0.4915	0.098*	
H5B	0.5213	0.4010	0.5960	0.098*	
H5C	0.4727	0.2333	0.5971	0.098*	
C6	0.62213 (15)	0.0496 (2)	0.6924 (2)	0.0692 (6)	
H6A	0.5651	0.0034	0.7039	0.104*	
H6B	0.6759	0.0151	0.7689	0.104*	
H6C	0.6298	0.0150	0.6037	0.104*	
C7	0.89734 (10)	0.2189 (2)	0.79953 (16)	0.0427 (4)	
H7	0.9106	0.2800	0.7226	0.051*	
C8	0.96785 (13)	0.2667 (3)	0.9330 (2)	0.0733 (6)	
H8A	0.9625	0.3801	0.9478	0.110*	
H8B	1.0306	0.2429	0.9282	0.110*	
H8C	0.9566	0.2083	1.0104	0.110*	
C9	0.90379 (17)	0.0463 (3)	0.7687 (3)	0.0834 (7)	
H9A	0.8915	−0.0166	0.8429	0.125*	
H9B	0.9664	0.0226	0.7633	0.125*	
H9C	0.8576	0.0206	0.6797	0.125*	
N1	0.80376 (9)	0.26201 (16)	0.80317 (13)	0.0398 (3)	
H1A	0.7925	0.2590	0.8840	0.048*	
O1	0.73426 (8)	0.26804 (15)	0.53446 (11)	0.0531 (3)	
P1	0.71920 (3)	0.31507 (5)	0.66763 (4)	0.03470 (12)	

### Atomic displacement parameters (Å^2^)

**Table:**

	*U*^11^	*U*^22^	*U*^33^	*U*^12^	*U*^13^	*U*^23^
C1	0.0490 (10)	0.0468 (9)	0.0619 (11)	0.0063 (8)	0.0167 (9)	0.0155 (9)
C2	0.1089 (19)	0.0465 (12)	0.1096 (19)	0.0121 (12)	0.0570 (16)	−0.0108 (12)
C3	0.0785 (15)	0.0565 (13)	0.112 (2)	−0.0137 (11)	0.0373 (14)	0.0151 (12)
C4	0.0398 (8)	0.0532 (10)	0.0348 (8)	−0.0039 (7)	0.0148 (7)	0.0047 (7)
C5	0.0366 (9)	0.0924 (16)	0.0629 (12)	−0.0074 (9)	0.0087 (8)	0.0160 (11)
C6	0.0697 (13)	0.0576 (12)	0.0814 (15)	−0.0150 (10)	0.0243 (12)	0.0030 (10)
C7	0.0377 (8)	0.0581 (11)	0.0365 (8)	0.0107 (7)	0.0174 (7)	0.0075 (7)
C8	0.0426 (10)	0.1149 (19)	0.0573 (12)	0.0060 (11)	0.0072 (9)	−0.0030 (12)
C9	0.0787 (15)	0.0704 (15)	0.1029 (19)	0.0283 (12)	0.0297 (14)	−0.0056 (13)
N1	0.0383 (7)	0.0593 (8)	0.0250 (6)	0.0094 (6)	0.0143 (5)	0.0085 (6)
O1	0.0553 (7)	0.0817 (9)	0.0270 (6)	0.0022 (6)	0.0193 (5)	0.0009 (5)
P1	0.03607 (19)	0.0451 (2)	0.02528 (19)	0.00340 (18)	0.01274 (14)	0.00474 (18)

### Geometric parameters (Å, °)

**Table:**

C1—C3	1.521 (3)	C6—H6A	0.9800
C1—C2	1.528 (3)	C6—H6B	0.9800
C1—P1	1.8150 (18)	C6—H6C	0.9800
C1—H1B	1.0000	C7—N1	1.4644 (19)
C2—H2A	0.9800	C7—C8	1.498 (3)
C2—H2B	0.9800	C7—C9	1.505 (3)
C2—H2C	0.9800	C7—H7	1.0000
C3—H3A	0.9800	C8—H8A	0.9800
C3—H3B	0.9800	C8—H8B	0.9800
C3—H3C	0.9800	C8—H8C	0.9800
C4—C6	1.515 (3)	C9—H9A	0.9800
C4—C5	1.527 (2)	C9—H9B	0.9800
C4—P1	1.8175 (16)	C9—H9C	0.9800
C4—H4	1.0000	N1—P1	1.6265 (14)
C5—H5A	0.9800	N1—H1A	0.8800
C5—H5B	0.9800	O1—P1	1.4799 (11)
C5—H5C	0.9800		
			
C3—C1—C2	111.54 (18)	H6A—C6—H6B	109.5
C3—C1—P1	109.54 (13)	C4—C6—H6C	109.5
C2—C1—P1	112.84 (13)	H6A—C6—H6C	109.5
C3—C1—H1B	107.6	H6B—C6—H6C	109.5
C2—C1—H1B	107.6	N1—C7—C8	109.72 (14)
P1—C1—H1B	107.6	N1—C7—C9	111.68 (15)
C1—C2—H2A	109.5	C8—C7—C9	112.11 (17)
C1—C2—H2B	109.5	N1—C7—H7	107.7
H2A—C2—H2B	109.5	C8—C7—H7	107.7
C1—C2—H2C	109.5	C9—C7—H7	107.7
H2A—C2—H2C	109.5	C7—C8—H8A	109.5
H2B—C2—H2C	109.5	C7—C8—H8B	109.5
C1—C3—H3A	109.5	H8A—C8—H8B	109.5
C1—C3—H3B	109.5	C7—C8—H8C	109.5
H3A—C3—H3B	109.5	H8A—C8—H8C	109.5
C1—C3—H3C	109.5	H8B—C8—H8C	109.5
H3A—C3—H3C	109.5	C7—C9—H9A	109.5
H3B—C3—H3C	109.5	C7—C9—H9B	109.5
C6—C4—C5	111.69 (16)	H9A—C9—H9B	109.5
C6—C4—P1	109.96 (12)	C7—C9—H9C	109.5
C5—C4—P1	110.96 (11)	H9A—C9—H9C	109.5
C6—C4—H4	108.0	H9B—C9—H9C	109.5
C5—C4—H4	108.0	C7—N1—P1	124.33 (10)
P1—C4—H4	108.0	C7—N1—H1A	117.8
C4—C5—H5A	109.5	P1—N1—H1A	117.8
C4—C5—H5B	109.5	O1—P1—N1	113.17 (7)
H5A—C5—H5B	109.5	O1—P1—C1	109.89 (8)
C4—C5—H5C	109.5	N1—P1—C1	108.11 (8)
H5A—C5—H5C	109.5	O1—P1—C4	113.05 (8)
H5B—C5—H5C	109.5	N1—P1—C4	104.85 (7)
C4—C6—H6A	109.5	C1—P1—C4	107.44 (8)
C4—C6—H6B	109.5		

### Hydrogen-bond geometry (Å, °)

**Table:**

*D*—H···*A*	*D*—H	H···*A*	*D*···*A*	*D*—H···*A*
N1—H1A···O1^i^	0.88	1.98	2.8344 (17)	165

Symmetry codes: (i) *x*, −*y*+1/2, *z*+1/2.
